# (*E*)-1,2-Bis(4-fluoro­phen­yl)ethane-1,2-dione

**DOI:** 10.1107/S1600536808023350

**Published:** 2008-07-31

**Authors:** Hoong-Kun Fun, Reza Kia

**Affiliations:** a X-ray Crystallography Unit, School of Physics, Universiti Sains Malaysia, 11800 USM, Penang, Malaysia

## Abstract

The title compound, C_14_H_8_F_2_O_2_, is a substituted benzil with an *s-trans* conformation of the dicarbonyl unit. This conformation is also shown by the O—C—C—O torsion angle of −110.65 (12)°. An unusual feature of the structure is the length, 1.536 (2) Å, of the central C—C bond connecting the carbonyl units, which is significantly longer than a normal C*sp* ^2^—C*sp* ^2^ single bond. This is probably the result of decreasing the unfavourable vicinal dipole–dipole inter­actions by increasing the distance between the two electronegative O atoms [O⋯O = 3.1867 (12) Å] and allowing orbital overlap of the dione with the π system of the benzene rings. The dihedral angle between the aromatic rings is 64.74 (5)°. In the crystal structure, neighbouring mol­ecules are linked together by weak inter­molecular C—H⋯O (× 2) hydrogen bonds. In addition, the crystal structure is further stabilized by inter­molecular π–π inter­actions with centroid–centroid distances in the range 3.6416 (6)–3.7150 (7) Å.

## Related literature

For bond-length data, see Allen *et al.* (1987 ▶). For carbonyl–carbonyl interaction, see Allen *et al.* (1998 ▶). For related structures and applications see, for example: Kaftory & Rubin (1983 ▶); Frey *et al.* (1995 ▶); Crowley *et al.* (1983 ▶); More *et al.* (1987 ▶); Brown *et al.* (1965 ▶); Gabe *et al.* (1981 ▶); Kimura *et al.* (1979 ▶); Stevens & Dubois (1962 ▶); Shimizu & Bartlett (1976 ▶); Rubin (1978 ▶).
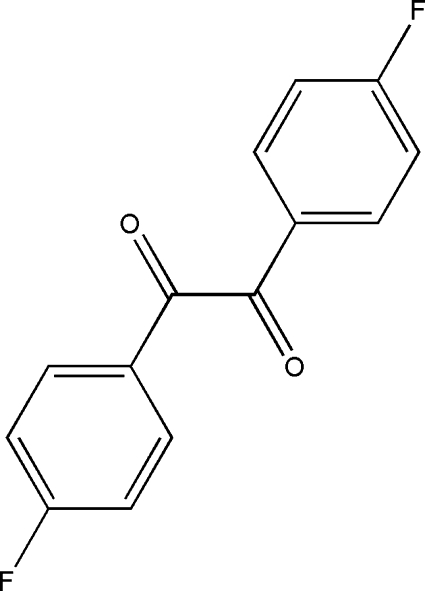


## Experimental

### 

#### Crystal data

C_14_H_8_F_2_O_2_
*M* *_r_* = 246.20
Monoclinic, 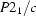

*a* = 12.1351 (2) Å
*b* = 7.3500 (1) Å
*c* = 13.1572 (2) Å
β = 110.507 (1)°
*V* = 1099.16 (3) Å^3^
*Z* = 4
Mo *K*α radiation
μ = 0.12 mm^−1^
*T* = 100.0 (1) K
0.39 × 0.30 × 0.28 mm

#### Data collection

Bruker SMART APEXII CCD area-detector diffractometer
Absorption correction: multi-scan (*SADABS*; Bruker, 2005 ▶) *T*_min_ = 0.903, *T*_max_ = 0.967
14891 measured reflections
3489 independent reflections
2846 reflections with *I* > 2σ(*I*)
*R*_int_ = 0.022

#### Refinement

*R*[*F* ^2^ > 2σ(*F* ^2^)] = 0.042
*wR*(*F* ^2^) = 0.107
*S* = 1.06
3489 reflections
163 parameters
H-atom parameters constrained
Δρ_max_ = 0.39 e Å^−3^
Δρ_min_ = −0.21 e Å^−3^

### 

Data collection: *APEX2* (Bruker, 2005 ▶); cell refinement: *APEX2*; data reduction: *SAINT* (Bruker, 2005 ▶); program(s) used to solve structure: *SHELXTL* (Sheldrick, 2008 ▶); program(s) used to refine structure: *SHELXTL*; molecular graphics: *SHELXTL*; software used to prepare material for publication: *SHELXTL* and *PLATON* (Spek, 2003 ▶).

## Supplementary Material

Crystal structure: contains datablocks global, I. DOI: 10.1107/S1600536808023350/zl2128sup1.cif

Structure factors: contains datablocks I. DOI: 10.1107/S1600536808023350/zl2128Isup2.hkl

Additional supplementary materials:  crystallographic information; 3D view; checkCIF report

## Figures and Tables

**Table 1:** Selected centroid⋯centroid distances (Å)

*Cg*1⋯*Cg*1^i^	3.6416 (6)
*Cg*2⋯*Cg*2^ii^	3.7150 (7)

Symmetry codes: (i) 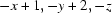; (ii) 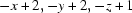. *Cg*1 and *Cg*2 are the centroids of the C1–C6 and C9–C14 benzene rings, respectively.

**Table 2:** Hydrogen-bond geometry (Å, °)

*D*—H⋯*A*	*D*—H	H⋯*A*	*D*⋯*A*	*D*—H⋯*A*
C2—H2*A*⋯O1^iii^	0.93	2.40	3.2648 (15)	155
C11—H11*A*⋯O2^iv^	0.93	2.51	3.3098 (16)	145

Symmetry codes: (iii) 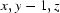; (iv) 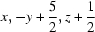.

**Fig. 1.**
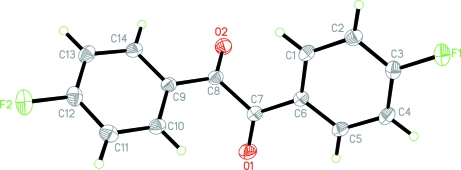
The molecular structure of (I) with atom labels and 50% probability ellipsoids for non-H atoms.

**Fig. 2.**
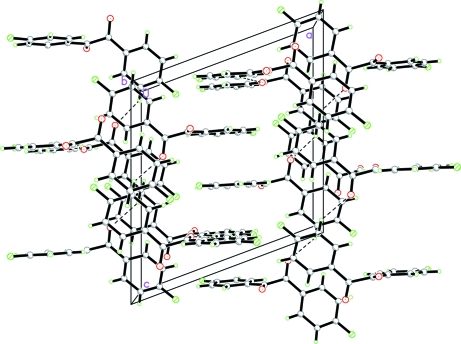
The crystal packing, showing parallel aligning of the benzene rings along the c axis. Intermolecular C—H···O interactions are shown as dashed lines.

## supplementary crystallographic information

### Comment

Investigation of photophysical properties of α-dicarbonyls has focused on the intramolecular carbonyl group electronic interaction as a function of their geometrical relationship. In previous extensive studies of the photochemistry of these compounds (Stevens & Dubois, 1962; Shimizu & Bartlett, 1976), biacetyl and benzil were the exclusive experimental vehicles for photophysical study. The structure of vicinal di- and polycarbonyl compounds have been of interest for many years (Rubin, 1978; Crowley *et al.*, 1983; Kaftory *et al.*, 1983; Frey *et al.*, 1995; Kimura *et al.*, 1979). Only a limited amount of data has been gathered from solid-state configurations such as in single crystals or as inclusion dopants in host crystals.

In the title compound (I, Fig.1), bond lengths, bond angles, and torsion angles of the dicarbonyl unit deviate significantly from normal values (Allen *et al.*, 1987) in order to minimize the repulsive interactions resulting from juxtaposition of dipolar carbonyl groups (Allen *et al.*, 1987). The C7–C8 bond distance connecting the carbonyl units is longer than those in normal *sp^2^*–*sp^2^* single bonds, such as in butadiene. This is probably the result of decreasing the unfavourable vicinal dipole-dipole interactions. The dicarbonyl unit has an *s-trans* conformation as indicated by the torsion angle of O1–C7–C6–C5, and O2–C8–C9–C10 being -174.32 (11) and 174.49 (11)°, respectively. This conformation is substantiated by the torsion angle of O–C–C–O, being -110.65 (12)°. The overal effect is to maximize the distance between the two electronegative oxygen atoms [O1···O2 = 3.1867 (12) Å] and to allow orbital overlap of the dione with the π system of the benzene rings. The dihedral angle between two phenyl rings is 64.74 (5)°. In the crystal structure, neighbouring molecules are linked together by weak intermolecular C—H···O (2 ×) hydrogen bonds. The packing mode (Fig. 2) tends to be dominated by van der Waals close packing interactions and the preference for aligning the substituted phenyl rings parallel to each other along the *c* axis with centroid to centroid distances of the π rings of 3.6416 (6) to 3.7150 (7) Å.

### Experimental

The synthetic method has been described earlier (Frey *et al.*, 1995). Single crystals suitable for *X*-ray diffraction were obtained by evaporation of a methanol solution at room temperature.

### Refinement

All of the hydrogen atoms were positioned geometrically and refined using a riding model with isotropic thermal parameters 1.2 times that of the parent atom.

### Crystal data

**Table:**

C_14_H_8_F_2_O_2_	*F*_000_ = 504
*M**_r_* = 246.20	*D*_x_ = 1.488 Mg m^−^^3^
Monoclinic, *P*2_1_/*c*	Mo *K*α radiation λ = 0.71073 Å
Hall symbol: -P 2ybc	Cell parameters from 6651 reflections
*a* = 12.1351 (2) Å	θ = 3.2–30.8º
*b* = 7.3500 (1) Å	µ = 0.12 mm^−^^1^
*c* = 13.1572 (2) Å	*T* = 100.0 (1) K
β = 110.507 (1)º	Block, pale-yellow
*V* = 1099.16 (3) Å^3^	0.39 × 0.30 × 0.28 mm
*Z* = 4	

### Data collection

**Table:**

Bruker SMART APEXII CCD area-detector diffractometer	3489 independent reflections
Radiation source: fine-focus sealed tube	2846 reflections with *I* > 2σ(*I*)
Monochromator: graphite	*R*_int_ = 0.022
*T* = 100.0(1) K	θ_max_ = 31.0º
φ and ω scans	θ_min_ = 3.2º
Absorption correction: multi-scan(SADABS; Bruker, 2005)	*h* = −15→17
*T*_min_ = 0.903, *T*_max_ = 0.967	*k* = −8→10
14891 measured reflections	*l* = −18→18

### Refinement

**Table:**

Refinement on *F*^2^	Secondary atom site location: difference Fourier map
Least-squares matrix: full	Hydrogen site location: inferred from neighbouring sites
*R*[*F*^2^ > 2σ(*F*^2^)] = 0.042	H-atom parameters constrained
*wR*(*F*^2^) = 0.107	*w* = 1/[σ^2^(*F*_o_^2^) + (0.0458*P*)^2^ + 0.3386*P*] where *P* = (*F*_o_^2^ + 2*F*_c_^2^)/3
*S* = 1.06	(Δ/σ)_max_ = 0.001
3489 reflections	Δρ_max_ = 0.39 e Å^−^^3^
163 parameters	Δρ_min_ = −0.21 e Å^−^^3^
Primary atom site location: structure-invariant direct methods	Extinction correction: none

### Special details

**Table:**

Experimental. The low-temperature data was collected with the Oxford Cyrosystem Cobra low-temperature attachment.
Geometry. All e.s.d.'s (except the e.s.d. in the dihedral angle between two l.s. planes) are estimated using the full covariance matrix. The cell e.s.d.'s are taken into account individually in the estimation of e.s.d.'s in distances, angles and torsion angles; correlations between e.s.d.'s in cell parameters are only used when they are defined by crystal symmetry. An approximate (isotropic) treatment of cell e.s.d.'s is used for estimating e.s.d.'s involving l.s. planes.
Refinement. Refinement of *F*^2^ against ALL reflections. The weighted *R*-factor *wR* and goodness of fit *S* are based on *F*^2^, conventional *R*-factors *R* are based on *F*, with *F* set to zero for negative *F*^2^. The threshold expression of *F*^2^ > σ(*F*^2^) is used only for calculating *R*-factors(gt) *etc*. and is not relevant to the choice of reflections for refinement. *R*-factors based on *F*^2^ are statistically about twice as large as those based on *F*, and *R*- factors based on ALL data will be even larger.

### Fractional atomic coordinates and isotropic or equivalent isotropic displacement parameters (Å^2^)

**Table:**

	*x*	*y*	*z*	*U*_iso_*/*U*_eq_	
F1	0.35237 (6)	0.66325 (10)	0.08245 (6)	0.03021 (18)	
F2	1.21050 (6)	1.20663 (11)	0.59266 (6)	0.03299 (19)	
O1	0.69635 (7)	1.33900 (11)	0.19570 (7)	0.0273 (2)	
O2	0.85192 (7)	1.05531 (12)	0.12153 (7)	0.02583 (19)	
C1	0.64396 (10)	0.85311 (15)	0.16653 (9)	0.0200 (2)	
H1A	0.7218	0.8152	0.1869	0.024*	
C2	0.55476 (10)	0.72510 (16)	0.14036 (9)	0.0217 (2)	
H2A	0.5712	0.6012	0.1434	0.026*	
C3	0.44035 (10)	0.78766 (16)	0.10962 (9)	0.0215 (2)	
C4	0.41085 (10)	0.96950 (17)	0.10511 (9)	0.0223 (2)	
H4A	0.3327	1.0060	0.0844	0.027*	
C5	0.50092 (10)	1.09585 (16)	0.13226 (8)	0.0202 (2)	
H5A	0.4836	1.2193	0.1304	0.024*	
C6	0.61811 (9)	1.03855 (15)	0.16255 (8)	0.0183 (2)	
C7	0.71259 (9)	1.17601 (15)	0.18983 (9)	0.0201 (2)	
C8	0.83778 (9)	1.11508 (14)	0.20254 (9)	0.0195 (2)	
C9	0.93465 (9)	1.14174 (14)	0.30709 (9)	0.0186 (2)	
C10	0.91694 (10)	1.22467 (15)	0.39561 (9)	0.0206 (2)	
H10A	0.8422	1.2644	0.3896	0.025*	
C11	1.01017 (11)	1.24806 (15)	0.49231 (10)	0.0231 (2)	
H11A	0.9995	1.3047	0.5514	0.028*	
C12	1.11907 (10)	1.18496 (16)	0.49832 (9)	0.0230 (2)	
C13	1.14030 (10)	1.09925 (16)	0.41347 (10)	0.0228 (2)	
H13A	1.2149	1.0563	0.4212	0.027*	
C14	1.04700 (9)	1.07948 (15)	0.31680 (9)	0.0204 (2)	
H14A	1.0589	1.0245	0.2578	0.025*	

### Atomic displacement parameters (Å^2^)

**Table:**

	*U*^11^	*U*^22^	*U*^33^	*U*^12^	*U*^13^	*U*^23^
F1	0.0221 (3)	0.0307 (4)	0.0367 (4)	−0.0068 (3)	0.0089 (3)	−0.0029 (3)
F2	0.0255 (4)	0.0401 (5)	0.0272 (4)	−0.0061 (3)	0.0014 (3)	0.0002 (3)
O1	0.0255 (4)	0.0194 (4)	0.0371 (5)	0.0035 (3)	0.0110 (4)	0.0006 (3)
O2	0.0237 (4)	0.0296 (4)	0.0263 (4)	−0.0006 (3)	0.0114 (3)	−0.0042 (3)
C1	0.0174 (5)	0.0227 (5)	0.0199 (5)	0.0039 (4)	0.0064 (4)	0.0032 (4)
C2	0.0225 (5)	0.0202 (5)	0.0227 (5)	0.0026 (4)	0.0082 (4)	0.0026 (4)
C3	0.0189 (5)	0.0256 (6)	0.0200 (5)	−0.0022 (4)	0.0068 (4)	−0.0002 (4)
C4	0.0169 (5)	0.0291 (6)	0.0207 (5)	0.0041 (4)	0.0062 (4)	0.0011 (4)
C5	0.0199 (5)	0.0220 (5)	0.0190 (5)	0.0051 (4)	0.0070 (4)	0.0014 (4)
C6	0.0179 (5)	0.0207 (5)	0.0166 (4)	0.0024 (4)	0.0065 (4)	0.0016 (4)
C7	0.0192 (5)	0.0216 (5)	0.0200 (5)	0.0023 (4)	0.0075 (4)	0.0014 (4)
C8	0.0189 (5)	0.0162 (5)	0.0251 (5)	0.0003 (4)	0.0097 (4)	0.0009 (4)
C9	0.0184 (5)	0.0148 (5)	0.0238 (5)	−0.0002 (4)	0.0089 (4)	0.0014 (4)
C10	0.0199 (5)	0.0183 (5)	0.0264 (5)	0.0001 (4)	0.0118 (4)	0.0011 (4)
C11	0.0272 (6)	0.0203 (5)	0.0243 (5)	−0.0031 (4)	0.0121 (4)	−0.0010 (4)
C12	0.0215 (5)	0.0212 (5)	0.0240 (5)	−0.0046 (4)	0.0050 (4)	0.0033 (4)
C13	0.0177 (5)	0.0213 (5)	0.0302 (6)	0.0005 (4)	0.0093 (4)	0.0045 (4)
C14	0.0207 (5)	0.0172 (5)	0.0263 (5)	0.0013 (4)	0.0118 (4)	0.0020 (4)

### Geometric parameters (Å, °)

**Table:**

F1—C3	1.3551 (13)	C6—C7	1.4751 (15)
F2—C12	1.3536 (14)	C7—C8	1.5358 (15)
O1—C7	1.2209 (14)	C8—C9	1.4764 (15)
O2—C8	1.2193 (13)	C9—C10	1.3962 (15)
C1—C2	1.3836 (16)	C9—C14	1.4009 (14)
C1—C6	1.3955 (15)	C10—C11	1.3854 (17)
C1—H1A	0.9300	C10—H10A	0.9300
C2—C3	1.3818 (15)	C11—C12	1.3764 (17)
C2—H2A	0.9300	C11—H11A	0.9300
C3—C4	1.3795 (17)	C12—C13	1.3824 (17)
C4—C5	1.3825 (16)	C13—C14	1.3826 (16)
C4—H4A	0.9300	C13—H13A	0.9300
C5—C6	1.4005 (14)	C14—H14A	0.9300
C5—H5A	0.9300		
			
Cg1···Cg1^i^	3.6416 (6)	Cg2···Cg2^ii^	3.7150 (7)
			
C2—C1—C6	120.58 (10)	O2—C8—C9	123.53 (10)
C2—C1—H1A	119.7	O2—C8—C7	116.53 (10)
C6—C1—H1A	119.7	C9—C8—C7	119.81 (9)
C3—C2—C1	117.70 (11)	C10—C9—C14	119.72 (10)
C3—C2—H2A	121.1	C10—C9—C8	122.02 (9)
C1—C2—H2A	121.1	C14—C9—C8	118.26 (9)
F1—C3—C4	118.28 (10)	C11—C10—C9	120.35 (10)
F1—C3—C2	118.08 (10)	C11—C10—H10A	119.8
C4—C3—C2	123.64 (11)	C9—C10—H10A	119.8
C3—C4—C5	118.03 (10)	C12—C11—C10	118.01 (10)
C3—C4—H4A	121.0	C12—C11—H11A	121.0
C5—C4—H4A	121.0	C10—C11—H11A	121.0
C4—C5—C6	120.24 (10)	F2—C12—C11	118.37 (11)
C4—C5—H5A	119.9	F2—C12—C13	118.00 (10)
C6—C5—H5A	119.9	C11—C12—C13	123.63 (11)
C1—C6—C5	119.80 (10)	C12—C13—C14	117.81 (10)
C1—C6—C7	120.96 (10)	C12—C13—H13A	121.1
C5—C6—C7	119.24 (10)	C14—C13—H13A	121.1
O1—C7—C6	123.95 (10)	C13—C14—C9	120.44 (10)
O1—C7—C8	117.05 (10)	C13—C14—H14A	119.8
C6—C7—C8	118.80 (9)	C9—C14—H14A	119.8
			
C6—C1—C2—C3	0.53 (16)	O1—C7—C8—C9	65.39 (14)
C1—C2—C3—F1	179.13 (9)	C6—C7—C8—C9	−119.59 (11)
C1—C2—C3—C4	−0.89 (17)	O2—C8—C9—C10	174.49 (11)
F1—C3—C4—C5	−179.60 (9)	C7—C8—C9—C10	−1.26 (15)
C2—C3—C4—C5	0.41 (17)	O2—C8—C9—C14	−6.17 (16)
C3—C4—C5—C6	0.42 (15)	C7—C8—C9—C14	178.08 (9)
C2—C1—C6—C5	0.26 (16)	C14—C9—C10—C11	0.97 (16)
C2—C1—C6—C7	−179.72 (10)	C8—C9—C10—C11	−179.71 (10)
C4—C5—C6—C1	−0.75 (15)	C9—C10—C11—C12	−0.93 (16)
C4—C5—C6—C7	179.23 (10)	C10—C11—C12—F2	−179.73 (10)
C1—C6—C7—O1	−174.32 (10)	C10—C11—C12—C13	−0.22 (17)
C5—C6—C7—O1	5.70 (16)	F2—C12—C13—C14	−179.18 (10)
C1—C6—C7—C8	11.03 (15)	C11—C12—C13—C14	1.31 (17)
C5—C6—C7—C8	−168.95 (9)	C12—C13—C14—C9	−1.24 (16)
O1—C7—C8—O2	−110.65 (12)	C10—C9—C14—C13	0.15 (16)
C6—C7—C8—O2	64.37 (13)	C8—C9—C14—C13	−179.20 (10)

Symmetry codes: (i) −*x*+1, −*y*+2, −*z*; (ii) −*x*+2, −*y*+2, −*z*+1.

### Hydrogen-bond geometry (Å, °)

**Table:**

*D*—H···*A*	*D*—H	H···*A*	*D*···*A*	*D*—H···*A*
C2—H2A···O1^iii^	0.93	2.40	3.2648 (15)	155
C11—H11A···O2^iv^	0.93	2.51	3.3098 (16)	145

Symmetry codes: (iii) *x*, *y*−1, *z*; (iv) *x*, −*y*+5/2, *z*+1/2.
